# Is Psychopathy a Mental Disorder or an Adaptation? Evidence From a Meta-Analysis of the Association Between Psychopathy and Handedness

**DOI:** 10.1177/14747049211040447

**Published:** 2021-10-04

**Authors:** Lesleigh E. Pullman, Nabhan Refaie, Martin L. Lalumière, DB Krupp

**Affiliations:** 1 School of Psychology, 6363 University of Ottawa, 136 Jean-Jacques Lussier, Ottawa, Ontario K1N 6N5, Canada; 2 Gordon S. Lang School of Business and Economics, 3653 University of Guelph, 50 Stone Rd. E., Guelph, Ontario N1G 2W1, Canada; 3 Department of Interdisciplinary Studies, 157777 Lakehead University, 500 University Ave., Orillia, Ontario L3V 0B9, Canada

**Keywords:** Psychopathy, life history, adaptation, mental disorder, handedness

## Abstract

Psychopathy has historically been conceptualized as a mental disorder, but there is growing evidence that it may instead be an alternative, adaptive life history strategy designed by natural selection. Although the etiology of mental disorders is not fully understood, one likely contributor is perturbations affecting neurodevelopment. Nonright-handedness is a sign of such perturbations, and therefore can be used to test these competing models. If psychopathy is a mental disorder, psychopaths should show elevated rates of nonright-handedness. However, an adaptive strategy perspective expects psychopaths to be neurologically healthy and therefore predicts typical rates of nonright-handedness. We meta-analyzed 16 studies that investigated the association between psychopathy and handedness in various populations. There was no difference in the rates of nonright-handedness between community participants high and low in psychopathy. Furthermore, there was no difference between psychopathic and nonpsychopathic offenders in rates of nonright-handedness, though there was a tendency for offenders scoring higher on the Interpersonal/Affective dimension of psychopathy to have lower rates of nonright-handedness, and for offenders scoring higher on the Behavioral dimension of psychopathy to have higher rates of nonright-handedness. Lastly, there was no difference in rates of nonright-handedness between psychopathic and nonpsychopathic mental health patients. Thus, our results fail to support the mental disorder model and partly support the adaptive strategy model. We discuss limitations of the meta-analysis and implications for theories of the origins of psychopathy.

## Introduction

Psychopathy—a syndrome characterized by antisocial, impulsive, manipulative, and callous behavior—has long been considered a mental disorder. As popularized by Cleckley (1941/1976), psychopathy was primarily thought to be an expression of trait-based personality deficits, such as “lack of remorse,” “untruthfulness and insincerity,” and “general poverty in major affective reactions,” as well as a few behavioral deficits, such as “inadequately motivated antisocial behaviour.” Making his position on the mental wellbeing of psychopaths plain, Cleckley (1941/1976) wrote “[i]n the attempt to arrive at an applicable conception, one consistent with the facts of our observation, I find it necessary first of all to postulate that the psychopath has a genuine and very serious disability, disorder, defect, or deviation” (pp. 367–368).

Many of the characteristics identified by Cleckley (1941/1976) were incorporated into the diagnostic criteria for Antisocial Personality Disorder (APD) in the second edition of the Diagnostic and Statistical Manual of Mental Disorders (DSM-II; American Psychiatric Association [APA], 1968). Over time, however, most of Cleckley's characteristics were removed from the diagnostic criteria of APD in favor of behavioral indicators that could be more reliably assessed and were thought to typify the disorder (APA, 1980). Many have argued that this resulted in a conceptual shift, whereby psychopathy and APD had become distinct clinical diagnoses (e.g., Kosson et al., 2006) while others have argued that, since psychopathy might be a dimensional construct, APD represents the lower or less severe end of the psychopathy continuum (Coid & Ullrich, 2010). Indeed, a diagnosis of APD is strongly related to the behavioral/antisocial dimension of psychopathy, but it is less strongly related to the interpersonal/affective traits of psychopathy, which are often considered the core features of psychopathy (Hare et al., 1991).

APD is still listed as a disorder in the most recent edition of the DSM, the DSM-5 (APA, 2013). A diagnosis of APD requires the individual to show a disregard for and violation of the rights of others, since age 15, as indicated by three or more of these features: (1) failure to conform to social norms with respect to lawful behavior, (2) deceitfulness, (3) impulsivity/failure to plan ahead, (4) irritability and aggressiveness, (5) reckless disregard for the safety of self or others, (6) consistent irresponsibility, and (7) a lack of remorse. A diagnosis of conduct disorder with onset before age 15 is also required, illustrating the developmental continuity of the disorder. The DSM-5 authors wrote that “[t]his pattern has also been referred to as *psychopathy, sociopathy,* or *dyssocial personality disorder*” (APA, 2013, p. 659)*,* suggesting that they considered APD and psychopathy to overlap greatly. The ICD-10 (World Health Organization, 1992) also includes a clinical diagnosis (dissocial personality disorder) listed as synonymous with both psychopathic personality disorder and antisocial personality disorder. The new ICD-11 reformed the personality disorders section but still includes considerations of many traits associated with psychopathy (e.g., callousness, lack of empathy; Farnam & Zamanlu, 2018).

By and large, the scientific and mental health practitioner communities share the same outlook on psychopathy. For instance, Hart and Hare (1996) wrote that “given the morbidity of psychopathy and its negative impact on society, it is difficult to imagine that any mental disorder, save perhaps schizophrenia, could be considered a greater public health concern” (p. 131). Lynam (1997) was equally clear when he wrote that “in the psychopathic child … we have an opportunity to observe the development of the disorder before it has had an opportunity to destroy its host” (p. 434). Yet, despite the widespread belief that psychopathy is a mental disorder, an alternative, evolution-minded perspective has been proposed: that psychopathy is instead a life history strategy of social exploitation maintained by negative frequency-dependent selection (e.g., Harpending & Sobus, 1987; Harris et al., 2001; Krupp et al., 2012; Mealey, 1995). According to this view, the risk taking, opportunistic, and callous behavior exhibited by psychopaths would have increased reproductive success in ancestral environments under certain conditions (such as a high ratio of cooperators to psychopaths). Consequently, these traits would have been favored by selection over successive generations.

This study concerns a meta-analysis of one testable hypothesis related to the debate concerning psychopathy as a mental disorder versus an alternative life history strategy: the extent to which psychopathic individuals are more or less likely to exhibit signs of neurodevelopmental perturbations compared to nonpsychopathic individuals. Neurodevelopmental perturbations are difficult to assess directly, but can be measured indirectly. Nonright-handedness (left-handedness, mixed-handedness) is a sign of neurodevelopmental problems (Brandler & Paracchini, 2014; Carlsson et al., 1992; Oh et al., 2009; Orsini & Satz, 1986; for a review, see Schmitz et al., 2017; c.f. Bishop, 1990). Therefore, this meta-analysis will focus on an accessible proxy for neurodevelopmental perturbations: the extent to which psychopaths are more likely to display nonright-handedness compared to nonpsychopathic individuals.

### What is a Mental Disorder?

From an adaptationist perspective, Wakefield (1992; see also Nesse & Stein, 2012) has usefully defined the concept of mental illness as a *harmful dysfunction*. *Dysfunction* refers to the failure of an internal mechanism (physical, cognitive, or affective) to perform a function for which it has been designed through natural selection to perform, while *harmful* refers to the consequences of this mechanism's failure that are deemed undesirable by social and cultural standards. Under this conceptualization, a syndrome of cognition and behavior would have to satisfy both criteria—that it is dysfunctional and harmful—to qualify as a mental disorder.

The precise cause of the specific internal dysfunction might vary depending on the mental illness in question, and there may be many such causes. However, there are some common features present among many mental disorders. Although the etiology of major mental disorders (the causes of dysfunctions) is not well-understood, it is most often assumed that mental illness stems from a combination of environmental, genetic, epigenetic, and even stochastic factors (Mitchell, 2015).

Extreme forms of psychopathy are almost certainly dysfunctional, as they would likely have been in ancestral environments. However, this is true of *any* adaptation: a bone can be too dense; a tail, too long; a feeling, too strong. So, to the extent that a trait has been under direct selection, we can expect a typical or average form that is adaptive (at least, historically, and judged only against the available alternatives), and this is what we would call an “adaptation.” Conversely, to the extent that this same trait is subject to variation in its development or operation, we can expect a distribution around this form that may be suboptimal—and often maladaptive at the extremes. These suboptimal forms are not, themselves, the adaptation in question, because they were not favored by natural selection. And if these forms are also harmful (sensu Wakefield, 1992), we would call them “disorders.” Thus, as we discuss below, the question remains whether psychopathy, *on average*, shows evidence of disorder or adaptation.

### Neurodevelopmental Perturbations

Neurodevelopmental perturbations are conditions that influence pre-, peri-, and postnatal brain development. These perturbations are most commonly considered environmentally caused but can also arise from genetic mutations or abnormalities. The causes of these perturbations can occur during gestation (e.g., maternal malnutrition, infection), labor/delivery (e.g., anoxia), infancy (e.g., malnutrition, illness), and adolescence (e.g., head injury), and are thought to influence the formation and functioning of critical brain structures. For example, increased risk of schizophrenia has been found to be associated with a number of causes potentially affecting neurodevelopment including maternal malnutrition (Susser et al., 1996), maternal infection (Brown et al., 2000a; Buka et al., 2001; cf. Grech et al., 1997), maternal stress (Van Os & Selten, 1998), premature birth (Jones et al., 1998), complications during labor and delivery (Verdoux et al., 1997), as well as childhood trauma (Bentall et al., 2012; see also Van Winkel et al*.*, 2008).

While not as extensively studied, neurodevelopmental perturbations have also been linked to other major mental illnesses. This includes such disorders as depression, anxiety, mania, and posttraumatic stress disorder (Brown et al., 2000b; Entringer et al., 2009; Sanches et al., 2008), as well as autism (Gore et al., 2014) and borderline personality disorder (Putnam & Silk, 2005).

Neurodevelopmental perturbations are difficult to assess directly. Not only are brain structures and functioning difficult to detect without specialized equipment, but identifying factors known to cause neurodevelopmental perturbations often requires an extensive maternal and childhood medical history. Nonetheless, these perturbations can be assessed indirectly by handedness.

A recent meta-analysis of over two million people estimates that, over a range of measures, approximately 10.6% of the population is left-handed (Papadatou-Pastou et al., 2020). The human population bias for right-handedness is thought to stem from selection pressures throughout evolutionary history. The human body shows both structural and functional asymmetries, which are largely genetically influenced (Brandler et al., 2013). Brain lateralization is one type of asymmetry and refers to specific neural functions being more dominant in different hemispheres of the brain. It is thought that this asymmetry evolved to avoid excessive duplication of neural circuitry (Levy, 1977) as well as to reduce interference across different brain functions (Rogers, 2000). Linguistic processing and complex fine motor skills, for example, are functions most dominantly performed by the left hemisphere of the brain. As the left hemisphere controls the right side of the body, right-hand dominance may be a byproduct of genetically determined functional asymmetry of brain lateralization for left hemisphere language processing and/or fine motor skills (Steele & Uomini, 2009).

Given the strong human bias for being right-handed, what causes nonright-handedness? The mechanisms by which handedness develops are not known, but they likely occur very early in life. Evidence suggests that simple genetic models, such as those of Annett (1967) and McManus (1985), do not adequately explain individual differences in handedness, as the amount of variability in handedness in childhood and adulthood due to additive genetic variation is small (Armour et al., 2014; de Kovel & Francks, 2019; Medland et al., 2009; Vuoksimaa et al., 2009). Likewise, common or shared environmental effects are also small. This leaves the great majority of the variance in handedness attributable to stochastic or “noisy” developmental processes (McManus, 2021; Mitchell, 2018). Similar to minor physical anomalies and other signs of neurodevelopmental perturbations, nonright-handedness has been found to be related to low birth weight, birth complications, and prenatal stress (Coren, 2012; see also de Kovel et al., 2019), as well as prenatal exposure to hormones (Titus-Ernstoff et al., 2003). These results suggest that disruption to critical brain structures during pre- and perinatal brain development is associated with nonright-handedness. Indeed, those with a major mental illness (e.g., schizophrenia) are more likely to be left-handed relative to healthy control samples (Hirnstein & Hugdahl, 2014), as well as those with other types of mental illness (e.g., depression; Dragovic & Hammond, 2005; Nowakowska et al., 2008; Webb et al., 2013). For an alternative view of left-handedness—as a product of frequency-dependent selection—see Faurie et al. (2016).

### Psychopathy as an Alternative Life History Strategy

The perspective of psychopathy as a mental disorder has not gone unchallenged. Based on game theoretic models, Harpending and Sobus (1987) hypothesized the existence of a cheating adaptation: a combination of genetically transmitted traits that would facilitate the fitness of those who expressed them, by being nonreciprocating agents within a given population. The authors suggested that, in humans, psychopathy is the phenotype for this cheating adaptation.

Mealey (1995) further expanded this adaptationist hypothesis, arguing that there are two pathways by which psychopathy can be expressed. Primary psychopathy is an obligate life history strategy associated with genetically transmitted genotypes that produce a constellation of personality traits and is maintained by negative frequency-dependent selection: The traits associated with psychopathy (e.g., callousness, manipulativeness, lack of remorse) increase fitness, so long as these traits are rare in the population (Nettle, 2009). Secondary psychopathy is a facultative life history strategy that is triggered by specific environmental conditions (Mealey, 1995). The traits associated with psychopathy emerge out of changing individual and environmental contingencies. Individuals will display the characteristics associated with psychopathy (e.g., manipulative behavior) when such traits are expected to promote fitness in the specific environmental conditions the individual finds themselves. While Mealey (1995) provided a promising theoretical perspective, there is little evidence to support this environmentally contingent form of psychopathy. For example, while aversive family environments generally predict conduct-disordered behavior in youth, they do not predict conduct-disordered behavior in youth who are high in callous/unemotional traits. Indeed, members of the latter group are more likely to participate in conduct-disordered behavior regardless of their family environment (e.g., Oxford et al., 2003; Wootton et al., 1997).

In their analysis of the causes of sexual assault, Lalumière et al. (2005) postulated that there are three pathways that can lead to sexual assault (and crime more generally). The first is called the young male syndrome (Wilson & Daly, 1985), in which young men use sexual coercion to gain access to mates because they are competitively disadvantaged in regard to resources and status and thus lack the ability to gain access to mates by legitimate means. This pathway is limited to adolescence and young adulthood, and the coercive behavior eventually ceases when they are able to compete for mates via legitimate means. The second pathway is a life-course-persistent pathway (Moffitt, 1993). Individuals in this pathway are characterized by disadvantaged social environments and neurodevelopmental perturbations. Similar to the young male syndrome, sexual assault occurs because these individuals are not able to compete for mates by legitimate means. Antisocial behavior (including sexual assault) as a result of this pathway is expected to persist throughout adulthood because the effects of neurodevelopmental perturbations and disadvantaged social environments are cumulative and long-lasting.

The third pathway is psychopathy. Similar to the life-course-persistent pathway, antisocial behavior (including sexual assault) as a result of this pathway is expected to persist throughout adulthood. Unlike the life-course-persistent pathway, these individuals are healthy and can compete for mates in prosocial ways, but use sexual coercion when the benefits of this behavior outweigh the potential costs. Similar to Mealey's (1995) conceptualization of primary psychopathy, psychopathy is considered an alternative life history strategy, maintained by negative frequency-dependent selection (Harris et al., 2001; Krupp et al., 2012; Lalumière et al., 2005; Lalumière & Seto, 1998). According to this view, the risk-taking, opportunistic, and callous traits of psychopaths increased their reproductive success in past environments by exploiting the trusting, cooperative individuals who form the majority of the population. Thus, psychopathy is not a mental disorder—despite the harm it causes others—as it is not related to the failure of an evolved psychological mechanism, but is instead an alternative strategy that has been selected for because of its positive effects on (largely direct or personal) fitness.

As previously discussed, neurodevelopmental perturbations are important to understanding the etiology of mental illness. Therefore, evidence showing a positive association between psychopathy and neurodevelopmental perturbations would support the mental disorder model, whereas a null or negative association would support the adaptive life history model. Broadly speaking, if psychopathy is a mental disorder, we would expect to find that psychopaths show greater signs of developmental distress, behavioral maladaptation, and reproductive depression, whereas we would not expect to find this if psychopathy is an adaptation. Of course, both models agree that psychopathy may be maladaptive in contemporary environments at the extreme—leaving highly psychopathic individuals incarcerated for the majority of their adult lives, say—but the adaptive life history model posits that this will not be true *on average*.

Thus far, psychopathy is not associated with elevated rates of developmental distress. For instance, Lalumière et al. (2001) found negative associations between psychopathy scores and two measures of neurodevelopmental perturbations: obstetrical problems (e.g., perinatal infection, fetal distress) and fluctuating asymmetry (small random deviations from perfect symmetry in structures that are meant to be symmetrical). Moreover, nonoffenders had a lower rate of fluctuating asymmetry compared to nonpsychopathic offenders, and there was no difference between nonoffenders and psychopathic offenders on fluctuating asymmetry. Similarly, Brazil et al. (2021) did not find differences in fluctuating asymmetry between university students scoring high and low on a measure of psychopathy. Finally, using structural equation modeling, Harris et al. (2001) found that although psychopathy and measures of neurodevelopmental perturbations, such as low IQ, are both associated with criminal behavior, they are not associated with each other.

Moreover, psychopaths behave selectively. They are more likely than other offenders to perpetrate instrumental, goal-directed crimes (i.e., robbery), rather than reactive, emotional ones (Cornell et al., 1996; Williamson et al., 1987). They are more likely than other offenders to engage in precocious and coercive sexual behavior and to target adults over children in their sexual offences (Harris et al., 2007b). And, unlike people diagnosed with other mental illnesses (Daly & Wilson, 1988; Harris et al., 2007a), psychopaths are no more likely than other offenders to harm their genealogical kin (Krupp et al., 2012; see also Harris et al., 2007b; Hilton et al., 2015). Lastly, while individuals with serious mental disorders tend to have fewer offspring (Keller & Miller, 2006), psychopathy scores among offender and community samples are positively associated with the number of children produced (Harris et al., 2007b; Lalumière et al*.*, 2001; Vachon et al., 2012; see also Pulkkinen et al., 2009).

Notably, there are many studies that have investigated structural and functional brain differences between psychopathic and nonpsychopathic individuals. Indeed, psychopaths have been found to have various structural brain differences compared to the general population (Birbaumer et al., 2005; Blair et al., 2005; Goyer et al., 1994; Kiehl et al., 2001; Raine et al*.*, 2004). However, as pointed out by Krupp et al. (2013), differences are not synonymous with deficits or dysfunctions. To the extent that psychopaths differ behaviorally from nonpsychopaths, neurological differences between them must necessarily exist, and may reflect either dysfunctional or (alternative) functional brain organization. In the case of handedness, clearer predictions can be made about psychopathy that are relevant to the mental disorder and alternative life history perspectives of the origin of psychopathy.

### Meta-Analytic Approach

Here, we examine the prevalence of nonright-handedness in adults who scored high versus low in psychopathic traits, in various settings. As stated above, our aim is to use handedness as a measure of neurodevelopmental problems in psychopathy, in the same way that depressed reproduction, low intelligence, obstetrical problems, fluctuating asymmetry, and violence against genealogical kin have been used to measure this (Brazil et al., 2021; Harris et al., 2001, 2007b; Krupp et al., 2012; Lalumière et al*.*, 2001; Vachon et al., 2012). Alone, any of these variables would be considered “noisy” measures of developmental distress. To the extent that there is consistency *across* them, however, provides increasing support for some models and decreasing support for others.

Most studies that have investigated psychopathy examined groups of people meeting some criterion for psychopathy versus those that do not. Therefore, handedness information is most often reported as the frequency (or mean level) of nonright-handedness in each group. An ideal group comparison to test the prediction from an adaptive life history perspective involves psychopathic individuals and those with a major mental illness (e.g., schizophrenia, bipolar disorder). If adaptive life history perspectives of psychopathy are supported, we would expect the psychopathic group to have lower rates of nonright-handedness compared to those with another major mental illness (matched on criminal history and other relevant variables). The mental disorder perspective, however, predicts no difference. Unfortunately, after the conclusion of the systematic review for this meta-analysis, we find that this comparison has not been addressed in past research.

However, we can still gain insights into the tenability of adaptive life history perspectives from examining other group differences. Some studies have compared noncriminal and nonclinical samples that differ in how they score on a measure of psychopathy. An adaptive life history perspective of psychopathy predicts no difference between the psychopathic and nonpsychopathic groups in rates of nonright-handedness, simply because this is indicative of healthy development among psychopaths, whereas the mental illness perspective predicts higher rates of nonright-handedness in the psychopathy group. This comparison provides a clean test of the two perspectives.

Another study design is to compare psychopathic versus nonpsychopathic offenders in a prison setting. There is a high frequency of mental illness in prison populations (Brink et al., 2001; James & Glaze, 2006). Furthermore, as categorized by Moffitt (1993), individuals who continue criminal behavior into adulthood (and are thus more likely to be in prison) are considered life-course-persistent offenders. These individuals are often characterized by impoverished social environments and neurodevelopmental perturbations, and thus may have higher rates of nonright-handedness (Moffitt & Caspi, 2001). Also important is the relationship between nonright-handedness and criminality more generally. Ellis (1990) reviewed the literature concerning handedness and crime and concluded that individuals who were prone to commit criminal behavior were more likely to be nonright-handed. While psychopathy was not considered by Ellis, based on the low prevalence of psychopathy, we would expect these samples to predominantly include nonpsychopaths. This evidence would suggest that persistent criminal behavior should be associated with nonright-handedness. Based on these findings, if an adaptive life history perspective of psychopathy is tenable, then we would expect psychopathic offenders to have lower levels of nonright-handedness compared to nonpsychopathic offenders. Conversely, the mental disorder perspective predicts no difference.

A final group comparison involves psychopathic and nonpsychopathic forensic patients (recruited from mental health facilities/hospitals). This is different from the comparison reported above. Both groups here are expected to have mental illnesses (by the fact that they are being treated at a forensic hospital), but one group also has psychopathy while the other does not. These patients may or may not have also committed a crime. Adaptive life history perspectives of psychopathy predict no difference between the two groups, while the mental illness perspective predicts higher rates of nonright-handedness in the psychopathy group, as a function of having a mental illness as well as psychopathy.

## Method

Inclusion criteria for this meta-analysis specified that (1) the study had to be written in English, (2) include an identifiable sample of predominantly adult (over the age of 18) male and/or female participants, (3) include at least 10 participants in each group, (4) use a validated measure of psychopathy, (5) use a measure of handedness, and (6) contain enough information to calculate Cohen's *d*.

Due to the uncertainty surrounding the distinction between psychopathy and APD (Coid & Ullrich, 2010; Kosson et al., 2006), this review only included studies that used a validated measure of psychopathy (e.g., PCL-R, Levenson Self-Report Psychopathy Scale, Psychopathic Personality Inventory). Studies that used the DSM or ICD criteria alone, or did not specify the measure that was used to define psychopathy, were not included in this review.

Furthermore, nonright-handedness was defined as left-handedness, mixed handedness, or ambidexterity, measured with self-reported hand preference, hand preference when writing, or validated handedness inventories. The measures and, if applicable, cut-off scores used to define psychopathy and handedness were also coded.

### Search for Studies

Online searches for studies examining the relationship between psychopathy and handedness were conducted through PsycInfo, Pubmed/Medline, Proquest Dissertation and Theses, Academic Search Complete, Scholars Portal, and Science Direct. The following search terms were used: (Psychopath* OR Sociopath* OR Dissocial* OR Dyssocial* OR “*Antisocial Personality*” OR *APD*) and (Handed* OR Ambidex* OR Lateral* OR Sinistral* OR Dextral*). Many online databases only search titles/abstracts and indexed keyboards, rather than searching the full-text of an article. This was the case for PsycInfo, Pubmed/Medline, and Proquest Dissertation and Theses. Therefore, the terms above were searched for in the abstract of the articles for these databases. Academic Search Complete, Scholars Portal, and Science Direct do allow for full-text searching, and therefore the first string of terms was searched in the abstract of the article, while the second string of terms was searched in the full-text of the article. This search strategy was used to facilitate finding articles that may discuss handedness within the article, but since it is not the main focus of the article these terms would not be included in the title or abstract. Furthermore, additional studies were found by searching Google Scholar, as well as reviewing the reference list of included studies (see Table 1). The search for relevant studies was completed using these databases on July 19, 2016, but we contacted authors whose publications found during the systematic search seemed to suggest that they had the information we needed (even if it was not included in the publication) after this date. It should be noted that more studies were originally identified that included other comparisons (e.g., psychopathic offenders vs. community control participants). However, after coding all studies, there were not enough studies that addressed these comparisons to compute a meta-analyzed effect size. Therefore, these studies were subsequently removed from our study list.

**Table 1.** Summary of Systematic Search of Literature.

Database	Number of articles found	Number of articles after duplicates across databases removed	Number of full-text articles available	Number of articles after first screen	Final number of articles
PsycInfo	422	323	319	45	3
PubMed/Medline	473	228	228	8	0
Proquest Dissertation and Theses	68	62	54	17	4
Academic Search Complete	1,016	788	788	69	3
Scholars Portal	1,294	603	603	95	5
Science Direct	1,000	864	864	91	4
Google Scholar/Reference Search	/	/	/	/	3
Authors who Provided Additional Study/Codeable Data	/	/	/	/	3
Totals:	4,273	2,868	2,856	325	25

### Coding Procedure

For each sample, a Descriptive Statistics Form was coded in order to account for characteristics of the sample and potential moderator variables. Each comparison between handedness and psychopathy within a study was also coded using the Effect Size Coding Form (Coding Manual available upon request). In many instances, the same dataset was used for multiple studies and there was partial or complete overlap of participants; when this occurred, the studies were considered part of the same sample. Each sample was assigned a study number (e.g., 10), and each study within the sample was assigned its own number following a decimal point (i.e., 10.1 and 10.2). The study with better quality data (e.g., largest sample size, provided M/SD over frequencies) was given the label of 0.1 (e.g., 10.1). The remaining studies in the sample were given consecutive labels (10.2 and 10.3) from newest to oldest. Only data coded from the study labeled 0.1 were included in the analyses (as any data from the other studies were considered overlapping with study 0.1). These other studies were still included in our study list as members of the same sample, but it should be noted that effect size information was not collected or analyzed from these studies (unless the study provided unique information, such as a new comparison group).

*Interrater reliability.* In order to conduct interrater reliability analyses, all of the studies were coded by two separate raters (three studies were also coded for practice). The interrater reliability for continuous variables was analyzed using absolute intraclass correlation coefficients (ICCs) based on a two-way mixed single measure design (Hallgren, 2012). ICC ratings of .40 are considered fair agreement, .60 good agreement, and .75 excellent agreement (Cicchetti, 1994). The interrater reliability for categorical variables was assessed using Cohen's kappa statistic (Cohen, 1960) as well as percent agreement. Guidelines for interpreting Cohen's kappa consider 0.21 to be fair agreement, 0.41 moderate agreement, 0.61 substantial agreement, and 0.81 almost perfect agreement (Landis & Koch, 1977).

Interrater reliability analyses were based on 15 samples and excluded the three practice cases (this includes studies that were subsequently removed from the study list due to too few studies addressing the comparison presented). Percent agreement ranged from 65% to 100% (*M* = 90%, *Mdn* = 94%). Cohen's Kappa statistic ranged from 0.55 to 1.00 for all categorical variables (*M* = 0.87, *Mdn* = 0.89), when Kappa could be computed. ICCs ranged from .80 to 1.00 for all continuous variables (*M* = 0.96, *Mdn* = 1.00). For the computed effect sizes, the two raters had a high rate of interrater agreement (ICC = .98). Discrepancies between raters were resolved by discussion between the two raters.

### Overview of Analyses

*Effect size.* Cohen's *d* was the effect size used in this meta-analysis. Cohen's *d* is a standardized measure of the magnitude of differences between two groups. For continuous variables, Cohen's *d* was calculated directly from variable means/standard deviations, using the pooled standard deviation (Cohen, 1988; Hasselblad & Hedges, 1995). For dichotomous variables, log odds ratios were calculated and then converted to Cohen’s *d* (Sánchez-Meca et al., 2003); 0.5 was added to each cell to allow for analyses when there were empty cells (Fleiss, 1994). When data on psychopathy and handedness were presented on continuous scales across an entire sample, the correlation (*r*) between psychopathy and handedness was converted to Fisher's Z, and then to Cohen's *d* (Cohen, 1988).

A positive *d* indicated that the psychopathic group presented with a higher rate of nonright-handedness compared to the comparison group; a negative *d* indicated that the psychopathic group presented with a lower rate of nonright-handedness compared to the comparison group. To provide some context for the effect sizes reported in this study, Hirnstein and Hugdahl (2014) reported a meta-analysis of 33 effect sizes comparing rates of nonright-handedness between schizophrenic patients and healthy controls: Patients with schizophrenia had higher rates of nonright-handedness than healthy controls, Odds Ratio = 1.55, 95% CI [1.25–1.93], which corresponds to a *d* of 0.24.

*Aggregation of findings.* Effect sizes across studies were aggregated using both fixed-effect and random-effects meta-analysis (Borenstein et al., 2009). These different meta-analysis models have different assumptions. Fixed-effect meta-analysis assumes that there is one “true” effect size across all of the studies, and any difference between studies is due to sampling error. Random-effects meta-analysis assumes that there is a distribution of “true” effect sizes and therefore captures the mean of this distribution. While the results of both meta-analysis models are presented, focus was placed on the fixed-effect model to interpret the results, as *T*^2^ (between-study variability component necessary for random-effects meta-analysis) is imprecise with a small number of studies (Schulze, 2007).

*Heterogeneity in effect sizes.* Cochran's *Q* statistic and the *I*^2^ statistic were used to assess the degree of heterogeneity across studies (Borenstein et al., 2009). The *Q* statistic is a statistical significance test for variability across studies. The *I*^2^ value is a measure of effect size for variability across studies that is due to true heterogeneity as opposed to chance. *I*^2^ values of 25% are considered low, 50% moderate, and 75% high (Higgins et al., 2003).

Following the conventions of Hanson and Bussière (1998), a finding was considered an outlier if the overall variability (*Q*) was statistically significant (*p* < .05) and a single extreme value (highest or lowest) accounted for more than 50% of the total variance. In this case, the results are reported with and without the outlying case. Outliers were not removed in analyses with only three samples, however.

Furthermore, studies that have unusually large weights in a meta-analysis can also unduly influence the results. A study weight in fixed-effect meta-analysis is calculated as the inverse of the variance, and the variance is calculated based on the total sample size as well as the dispersion of the sample size across groups. However, if a single study has a substantially larger sample size than all of the other studies, the majority of the weight will be given to this single study, and the aggregated effect will essentially ignore all of the other studies. While this large study should be given more weight, it should not be the only consideration. Therefore, following conventions proposed by Babchishin and Helmus (2012), a single study was considered influential if the overall variability (*Q*) has a *p* value < .05, and the largest study weight was more than 50% larger than the next highest study weight. In these cases, the solution is to reduce the weight of the influential study to 50% larger than the next highest study weight. Results are reported for the original study weight as well as the reduced study weight, with interpretation of the results focusing on the effect size that includes the reduced study weight.

*Publication bias.* Publication bias is a threat to the validity of most meta-analyses (Souza et al., 2007) and refers to the tendency for studies that find statistically nonsignificant results to be less likely to be submitted or accepted for publication. In this meta-analysis, 81% of the samples were from published studies (*k* = 13) and 19% were from unpublished studies (*k* = 3); therefore, our sample is biased toward published studies. However, given that the relationship between handedness and psychopathy is rarely the focus of an article, but reported when describing the sample, the results may not be influenced by publication bias.

Egger's regression (Egger et al., 1997) was used to investigate the influence of publication bias. Egger's regression is a test of the asymmetry of the standard error by effect size funnel plot. Publication bias is evident when this plot does not follow the standard inverse V funnel pattern (see Borenstein et al., 2009). Due to the low statistical power of these tests and our small sample size, evidence of asymmetry in the funnel plot was indicated when *p* < .10 (Egger et al., 1997).

## Results

In total, 16 (*k*) nonoverlapping comparisons (from 25 individual reports) contributed to this meta-analysis (total *n* = 1,818). These reports were published or made available to use between 1985 and 2017 (*Mdn* = 2009). The psychopathic group was from a correctional setting (e.g., prison, parole) in 38% of the comparisons (*k* = 6), the general population (e.g., university sample, community samples) in 44% of the comparisons (*k* = 7), and a hospital or mental health facility in 19% of the comparisons (*k* = 3). In 56% of the comparisons (*k* = 9), the psychopathic group was being held in a secure custody setting. The unweighted average age of all participants across all comparisons was 29.2 years old (*SD* = 8.0). In 63% of the comparisons (*k* = 10), the participants were all men, whereas in 6% of the comparisons (*k* = 1), the participants were all women. In the remaining 31% of the comparisons (*k* = 5), there was a mix of men and women (the proportion of women ranged from 14% to 70% of the total sample in these comparisons). See Table 2 for more descriptive characteristics of the samples.

**Table 2.** Descriptive Summary of the Samples.

Study no.	Authors	Country	Percent male	Psychopathy measure	Handedness measure
1	Carolan et al. (2014)	Canada	38%	Psychopathic Personality Inventory—Short Form (Lilienfeld & Widows, 2005)	Unknown
2	Eisenbarth et al. (2008)	Italy	0%	Psychopathy Checklist—Revised (Hare et al., 1990)	Unknown
3	Ermer et al. (2012)	United States	100%	Psychopathy Checklist—Revised (Hare, 2003)	Self-report
4	Folsom (1993)	Canada	100%	Psychopathy Checklist—Revised (Hare, 1991)	Lateral Preference Questionnaire (Porac & Coren, 1981)
5.1 5.2 5.3	Hare & Forth (1985) Forth (1992) Hare et al. (1980)	Canada	100%	Psychopathy Checklist (Hare, 1980)	Lateral Preference Questionnaire (Porac & Coren, 1981)
6	Kosson et al. (1997)	United States	100%	Psychopathy Checklist—Revised (Hare, 1991)	Handedness Inventory (Chapman & Chapman, 1987)
7	Lalumière et al. (2001)	Canada	100%	Psychopathy Checklist—Revised (Hare, 1991)	Observed handwriting preference
8	Long & Titone (2007)	Unknown	100%	Psychopathic Personality Inventory—Short Form (Lilienfeld & Andrews, 1996)	Unknown
9	Mahmut & Stevenson (2016)	Australia	49%	Self-Report Psychopathy Scale (Paulhus et al., 2015)	Self-report
10.1 10.2	Mayer & Kosson (2000) Miller (2001)	United States	100%	Psychopathy Checklist—Revised (Hare, 1991)	Handedness Inventory (Chapman & Chapman, 1987)
11	O'Connor Pennuto (2007)	United States	100%	Psychopathy Checklist—Revised (Hare, 2003)	Handedness Inventory (Lezak, 1995; modified from Annett, 1967)
12	Pasion et al. (2016)	Portugal	40%	Triarchic Psychopathy Measure (Patrick, 2010)	Unknown
13	Salim et al. (2015)	Netherlands	100%	Triarchic Psychopathy Measure (Patrick, 2010)	Unknown
14	Shobe & Desimone (2016)	United States	28%	Short Dark Traid Scale—Psychopathy Subscale (Jones & Paulhus, 2014)	Edinburgh Handedness Inventory (Oldfield, 1971)
15	Stankovic et al. (2015)	Serbia	100%	Self-Report Psychopathy Scale-III (Williams et al., 2007)	Edinburgh Handedness Inventory—Short Form (Veale, 2014)
16.1 16.2 16.3 16.4 16.5 16.6 16.7	Yang et al. (2009) Glenn et al. (2010a) Glenn et al. (2010b) Yang et al. (2010) Yang et al. (2005) Raine et al. (2004) Raine et al. (2003)	United States	86%	Psychopathy Checklist—Revised (Hare, 2003)	Edinburgh Handedness Inventory (Oldfield, 1971)

*Note*. Miller (2001) overlaps with both Mayer and Kosson (2000) and Kosson et al. (1997). However, Mayer and Kosson (2000) and Kosson et al. (1997) do not overlap. Therefore, Miller (2001) was grouped with Mayer and Kosson (2000), with information being coded from Mayer and Kosson (2000) only. Kosson et al. (1997) was treated as an independent sample.

### High Versus Low Psychopathy in Community Samples

There was no difference between community participants high in psychopathic traits versus low in psychopathic traits in rates of nonright-handedness, *d* = 0.14, 95% CI [−0.04, 0.32], *I*^2^ = 58.0%, *k* = 7. The study weights within these fixed-effect results varied between 1.24 and 83.33 (*Mdn* = 2.28). The largest weight came from a study with a sample size of 342. This study had more than 4 times the weight of the next largest study weight, and more than 67 times the weight of the smallest study weight. To reduce the influence of this study, we reduced the study weight to be 50% larger than the next largest study weight. There was still no difference between community participants high in psychopathic traits versus low in psychopathic traits in rates of nonright-handedness, *d* = 0.01, 95% CI [−0.24, 0.26], *I*^2^ = 51.0%, *k* = 7 (weight reduced results; Table 3). There were no outliers identified in this aggregated effect, but there was still a moderate degree of variability in this effect size. Additionally, there was no evidence of publication bias for these effect sizes.

**Table 3.** Relationship Between Psychopathy and Handedness in Three Comparisons.

Comparison	Fixed-effect		Random-effects		*Q*	*I*^2^ (%)	*n* (*k*)	Studies contributing to effect size
	*d*	[95% CI]	*d*	[95% CI]				
High vs. Low Psychopathy in Community Samples	0.138	[−0.044, 0.321]	−0.077	[−0.531, 0.377]	14.30*	58.0	757 (7)	1, 8, 9, 12, 13, 14, 16.1
High vs. Low Psychopathy in Community Samples—Weight of influential study reduced	0.014	[−0.235, 0.263]	−0.075	[−0.522, 0.373]	12.23	51.0	757 (7)	1, 8, 9, 12, 13, 14, 16.1
Psychopathic vs. Non-Psychopathic Offenders	0.094	[−0.045, 0.232]	0.096	[−0.107, 0.299]	9.49	47.3	966 (6)	3, 4, 5.1, 6, 10.1, 11
Psychopathic vs. Non-Psychopathic Mental Health Patients	−0.097	[−0.804, 0.610]	−0.093	[−0.861, 0.674]	2.36	15.1	95 (3)	2, 7, 15

*Note.* A positive *d* indicates that psychopathic individuals presented with a higher rate of nonright-handedness than the comparison group; a negative *d* indicates that psychopathic individuals presented with a lower rate of nonright-handedness than the comparison group.

* *p* < .05.

** *p* < .01.

*** *p* < .001.

### Psychopathic Versus Nonpsychopathic Offenders

There were no differences between psychopathic and nonpsychopathic offenders in rates of nonright-handedness, *d* = 0.09, 95% CI [−0.05, 0.23], *I*^2^ = 47.3%, *k* = 6 (Table 3). There were no outliers identified in this aggregated effect, but there was still a moderate degree of variability. Additionally, there was no evidence of publication bias.

### Psychopathic Versus Nonpsychopathic Mental Health Patients

There were no differences between rates of nonright-handedness among psychopathic versus nonpsychopathic mental health patients, *d* = −0.10, 95% CI [−0.80, 0.61], *I^2^* = 15.1%, *k* = 3 (Table 3). There were no outliers identified, and little variability evident in this aggregated effect. Additionally, there was no evidence of publication bias.

### Factors of Psychopathy

Five studies examined the correlation between handedness and different factors of psychopathy (Factor 1: interpersonal/affective; Factor 2: behavioral). Four of these studies used an offender sample to examine this relationship, which allowed us to examine the relationship between handedness and different factors of psychopathy within offender samples (Table 4). The relationship between handedness and Factor 1 psychopathy (*d* = −.27, 95% CI [−0.55, 0.0005], *I*^2^ = 0.2%, *k* = 3; outlier removed results) suggests that, although this confidence interval does (barely) include zero, offenders who are higher on the interpersonal/affective dimension of psychopathy exhibited lower rates of nonright-handedness compared to offenders low on this dimension. Furthermore, the relationship between handedness and Factor 2 psychopathy (*d* = 0.16, 95% CI [−0.00009, 0.32], *I*^2^ = 0.0%, *k* = 4), suggest that, although the confidence intervals for this effect size includes (barely) zero as well, rates of nonright-handedness were higher among individuals who are high on the behavioral dimension of psychopathy compared to offenders low on this dimension.

**Table 4.** Relationship Between Handedness and Psychopathy Factors for Psychopathic Versus Non-Psychopathic Offenders.

	Fixed-effect		Random-effects		*Q*	*I*^2^ (%)	*n* (*k*)	Studies contributing to effect size
	*d*	[95% CI]	*d*	[95% CI]				
Factor 1: Interpersonal/Affective	0.103	[−0.056, 0.263]	−0.089	[−0.500, 0.322]	12.93**	76.8	626 (4)	4, 6, 10.1, 11
Factor 1: Interpersonal/Affective—outlier removed	−0.274	[−0.549, 0.0005]	−0.274	[−0.550, 0.0008]	2.00	0.2	206 (3)	4, 6, 11
Factor 2: Behavioral	0.159	[−0.00009, 0.319]	0.159	[−0.00009, 0.319]	2.93	0.0	626 (4)	4, 6, 10.1, 11

*Note.* A negative *d* indicates that individuals who had a higher scores on a given factor of psychopathy presented with a lower rate of nonright-handedness than those who scored lower on that factor. Conversely, a positive *d* indicates that individuals who had a higher score on a given factor presented with a higher rate of nonright-handedness than those who scored lower on that factor.

* *p* < .05.

** *p* < .01.

*** *p* < .001.

## Discussion

The mental disorder model of psychopathy received no support in this study. Psychopaths (or people scoring higher on psychopathy) did not have higher rates of nonright-handedness than did nonpsychopaths (or people scoring low on psychopathy). A qualification to this conclusion is necessary, however: the interpretation of the results very much depends on the nature of the sample being compared. In the psychopathic offender versus nonpsychopathic offender comparison, if the psychopaths do not have other clinical conditions and the nonpsychopathic offenders do, then the lack of difference in handedness would be consistent with the mental illness perspective. In the psychopathic versus nonpsychopathic mental health patients comparison, if the psychopathic patients do not have other clinical conditions and the nonpsychopathic patients do, then the lack of difference would also be consistent with a mental illness perspective. There were not enough details within the available studies to draw conclusions about these sample compositions.

Conversely, the results of this meta-analysis provide evidence both for and against an adaptive life history model of psychopathy. As predicted by this model, there was no difference in the rate of nonright-handedness between community participants high versus low on psychopathy. Although there was no difference between psychopathic versus nonpsychopathic offenders in rates of nonright-handedness (inconsistent with an adaptive life history perspective, if the nonpsychopaths in these studies have mental disorders), when we examined the relationship between handedness and the two factors of psychopathy within this group, we found that there was a tendency for offenders with higher scores on the interpersonal/affective dimension of psychopathy—considered the core features of psychopathy—having lower rates of nonright-handedness, whereas participants with higher scores on the behavioral dimension of psychopathy (perhaps conceptually more similar to APD and life-course-persistent offending) having higher rates of nonright-handedness, relative to their comparison groups. Lastly, and consistent with adaptive life history perspectives, there were no differences between psychopathic and nonpsychopathic mental health patients (if the two groups both have mental disorders other than psychopathy).

The most pertinent comparison for examining the validity of adaptive life history perspectives is the comparison between psychopathic individuals (with no comorbid mental illness) and those with a major mental illness. While all attempts were made to find articles that compared these two groups, we did not find any article that fit these criteria. Separately, however, Webb et al. (2013) found that 40% of patients with schizophrenia and schizoaffective disorder were left-handed, as measured by writing hand (a common measure of handedness), whereas Lalumière et al*.* (2001) found that only 8% of psychopathic offenders were left-handed, using the same measure. This is slightly below the population prevalence of left-handedness (Papadatou-Pastou et al., 2020). Moreover, Carolan et al. (2014) found that 6% and Salim et al. (2015) found that 0% of individuals from their university sample who were high in psychopathy and exhibited no comorbid major mental illness (via self-report) were left-handed. Future examinations of the validity of adaptive life history models of psychopathy (vs. mental disorder models) should focus on this specific comparison. Another and perhaps even more relevant contrast would be comparing psychopathic offenders with nonpsychopathic offenders, with both groups not having any other clinical conditions. However, there was not enough information included about the offender samples to conclude that they did not have any other clinical conditions.

There were a number of limitations to the current study. First, the number of primary studies that investigated the relationship between psychopathy and handedness was small. This increases the size of the confidence intervals and make the conclusions more tentative. This is particularly important in the context of hypotheses that involve no group differences: low statistical power prevents the discovery of group differences that could be there. Additionally, because of the small number of studies, a number of important moderator variables could not be assessed (e.g., the scale used to measure psychopathy, the cut-off score used to define psychopathic vs. nonpsychopathic individuals, mental illness status). Although we did investigate the relationship between handedness and different factors of psychopathy for offenders, this was not possible for the other sample types.

Second, we were not able to address the confounding effect of comorbidity. The rate of mental illness comorbidity with psychopathy is lower than that of other mental illnesses (Biji et al., 1998; cf. Blackburn et al., 2003), something that is consistent with an adaptive life history perspective. Nonetheless, it is still possible for psychopathic individuals to have another mental illness which could confound these results. Two of the samples (13%) included a group of psychopathic offenders who had a comorbid mental illness, whereas two of the samples (13%) only included participants without a comorbid mental illness. In the majority of samples (75%), it was not specified if the psychopathic group contained individuals who also had another mental illness. Given the small number of studies that reported this information, we were not able conduct moderator analyses for this variable.

Third, many of the samples in this meta-analysis were either partially or completely composed of female participants. Indeed, five samples (31%) had a mixed gender composition, and one sample (6%) had a completely female composition. The adaptive life history perspective of psychopathy presented here, however, pertains specifically to men (Lalumière et al., 2005). Therefore, the predictions it sets forth may not apply to women, and thus the mixed and exclusively female compositions of some samples may not be a good test of this theory. The comparison of psychopathic versus nonpsychopathic offenders includes studies that only sampled male participants, and thus is a more appropriate test of an adaptive life history perspective. Unfortunately, removing female samples from the comparison between community participants high versus low in psychopathy, as well as from the comparison between psychopathic versus nonpsychopathic forensic patients, would result in too few samples to conduct the analyses. For future examinations of this topic, results relating specifically to the relationship between psychopathy and handedness in men only may be more fruitful to assess the validity of the adaptive life history model.

Fourth, another potential confounding variable is related to the setting that the sample came from (e.g., university students/prison), as it relates to the measure of psychopathy used in the study. Indeed, in the comparison between community participants high and low in psychopathy, six of the seven studies used a measure of psychopathy other than the PCL-R, which is to be expected given the time commitment for PCL-R interviews. However, the remaining comparisons presented (psychopathic vs. nonpsychopathic offenders, psychopathic vs. nonpsychopathic forensic patients) were primarily composed of studies that used the PCL-R to define psychopathy. Because of the inability to conduct moderator analyses, it is unknown whether this confounding of setting and measure of psychopathy influenced the results.

Fifth, the current study defined nonright-handedness as left-handedness, mixed handedness, and ambidexterity. Whereas left- and right-handed individuals show patterns of dominance with their preferred hand when completing various tasks, mixed handedness and ambidexterity refers to a reduced degree of hand dominance. Although it was not possible to do so in this study, the *degree* of handedness would also be an important measure to investigate, particularly because it is more variable than, and confers information independent of, the direction of handedness (Edlin et al., 2015; Prichard et al., 2013). For example, two recent studies using convenience samples have found small, negative correlations between degree of handedness and psychopathy (Shobe & Desimone, 2016; Prichard, 2019), and it would be useful to study this further with larger samples, especially those drawn from populations with higher rates of psychopathy (e.g., clinical and prison populations).

Sixth, some researchers have differentiated between unsuccessful (incarcerated) and successful (nonincarcerated) psychopaths, hypothesizing that unsuccessful psychopaths suffer from cognitive impairments that lead them to criminal behavior to achieve their goals, whereas successful psychopaths actually have superior neurobiological functioning and are able to function within the law to achieve their selfish goals (Gao & Raine, 2010). It may be possible then that the comparison between psychopathic and nonpsychopathic offenders (all unsuccessful) in this meta-analysis would be expected to show no difference between groups, if unsuccessful psychopaths are indeed neurocognitively impaired. Indeed, the relationship between nonright-handedness and criminality more generally has been established (Ellis, 1990). This hypothesis would require further investigation.

Lastly, as with psychopathy, there may be negative frequency-dependent selection on handedness (Faurie et al., 2016). To our knowledge, no one has hypothesized that handedness coevolves with psychopathy—though this is certainly plausible, to the extent that both nonright-handedness and psychopathy are favored under conditions of violence (Billiard et al., 2005; Faurie & Raymond, 2005). Accordingly, this hypothesis makes much the same prediction as the mental disorder hypothesis: that psychopathy will be positively associated with the frequency of nonright-handedness. Our results, however, do not corroborate this hypothesis.

There remain a number of unanswered questions regarding adaptive life history versus mental illness perspectives of psychopathy. In addition to the need for more studies addressing the potential moderator variables discussed above, other testable hypotheses should be investigated. For example, studies directly comparing those with psychopathic traits (and no comorbid mental illness) to those who have a mental illness on various measures of neurodevelopmental perturbations, and studies investigating various ways in which psychopathy may enhance or reduce fitness will contribute to this literature. As an illustration, Krupp et al. (2012) found that psychopaths tend to offend against individuals with whom they are not biologically related, reducing indirect fitness costs.

A further research question concerns the distinction between psychopathy and APD. As suggested by Lalumière et al. (2005), a large portion of life-course-persistent offenders would meet the diagnostic criteria for APD, but only a small fraction of those would meet the criteria for psychopathy. We also know that, as a group, life-course-persistent offenders are characterized by aversive family environments and neurodevelopmental perturbations, while psychopaths, in particular, may not be affected by these factors (e.g., Lalumière et al*.*, 2001; Oxford et al., 2003). Mealey's (1995) postulation that secondary psychopathy is a contingent life history strategy born out of environmental pressure has not received much support yet, but it may be the case that APD is actually the expression of secondary psychopathy. The aversive environments experienced by these individuals may trigger the expression of genes associated with these traits. Mealey further suggested that secondary psychopathy is characterized more by antisocial behavior and less by the affective facets common among primary psychopathy (i.e., glibness), which mirrors our current conceptualization of the difference between psychopathy and APD. While primary psychopathy is an obligate strategy, secondary psychopathy is only expressed if environmental conditions (e.g., in utero) trigger these traits because such a response contributed to fitness over our evolutionary history. If this scenario proves tenable, then it may be that these two forms of psychopathy have different etiological origins.
